# Acinetobacter baumannii—The New MRSA?

**Published:** 2016-03-12

**Authors:** Alasdair Barrie, Mark Gorman

**Affiliations:** Department of Plastic Surgery, Royal Devon and Exeter Hospital, Exeter, United Kingdom

**Keywords:** *Acinetobacter*, transverse rectus abdominis myocutaneous, infection, breast, MRSA

**Figure:**
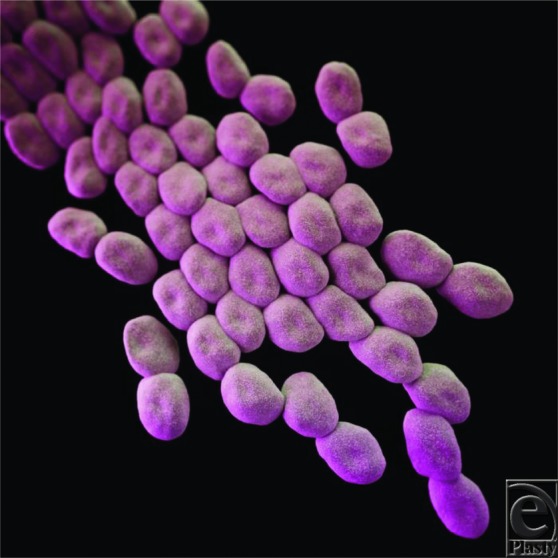


## DESCRIPTION

A 56-year-old woman with previous breast cancer underwent a muscle-sparing transverse rectus abdominis myocutaneous flap to her left breast. She developed marked flap swelling postoperatively and episodes of pyrexia.

A breast wound swab grew *Escherichia coli* and *Acinetobacter baumannii*. This strain of *Acinetobacter* was multidrug resistant, including resistance to meropenem.

## QUESTIONS

1. **What is *Acinetobacter* and how common is *Acinetobacter* infection?**
2. **How dangerous is *Acinetobacter* infection?**
3. **How is it diagnosed?**
4. **What precautions do you need to take and how do you treat it?**

## DISCUSSION

*Acinetobacter* is a pleomorphic aerobic gram-negative bacillus, increasingly found in hospitalized patients. Isolates from patients usually reflect colonization as opposed to infection. However, when the organism is pathogenic, it can be multidrug resistant and therefore difficult to treat. *Acinetobacter* infections rarely occur outside of health care settings but may occur in tropical environments, wars, and natural disasters. Its natural environment includes water and soil. In the hospital setting, it is most commonly found on the intensive care unit and burns units. In human populations, it colonizes the respiratory and gastrointestinal tracts, skin, and wounds. Some strains can survive in iron-deficient and dry environments for months, leading to the increased risk of fomite contamination.^1^ It is estimated to be the cause of 2% to 10% of all gram-negative infections in intensive care units in both the United States and Europe.^2^

*Acinetobacter* has low virulence but can cause infection in those who are immunocompromised, have chronic disease, have surgical wounds, or have prolonged hospital admissions. Infections caused by *Acinetobacter* can include bacteremia, hospital-acquired pneumonia, meningitis, and urinary tract infections.^3^ It can contaminate traumatic and surgical wounds, causing soft-tissue infection and osteomyelitis. Infections from *Acinetobacter baumannii* are renowned for being difficult to treat due to its antimicrobial resistance. This is in part due to its effectiveness in acquiring genetic material from other organisms and therefore rapidly developing drug resistance.^4^ A number of strains incorporate a polysaccharide capsule that may stop complement activation and protect against phagocytosis from macrophages.^5^ The species also form biofilms to adhere to environmental surfaces.^6^

Diagnosis can be achieved through taking appropriate samples for culture such as wound swabs or blood. In outbreaks, it is easy to culture *Acinetobacter* from monitors and equipment around the colonized patient. It is therefore paramount to thoroughly clean the ward and operating room the patient has been in. Microbiologically, it is nonmotile, catalase-positive, and oxidase-negative. In standard cultures, it appears as broad, short, gram-negative rods in rapid growth phase but a more coccobacillary shape in stationary phase. They do not reduce nitrate or ferment glucose.^7^

Those proven to be colonized with *Acinetobacter* should be isolated and appropriate infection control measure put in place. Supportive care includes removing or replacing any lines, catheters or drains. Regular discussion with Microbiology needs to take place to define colonization versus infection, as well as appropriate antibiotic regimens. Antibiotics used include meropenem, tigecycline, minocycline, colistin, amikacin, rifampin, and polymyxin B. Combination therapy may be required. Most infections, until recently, were sensitive to the carbapenems, but cases of resistance have been increasing. Multidrug-resistant *Acinetobacter* is commonly susceptible to antiseptic and disinfectants. Reports of failure to contain the bacteria are more likely due to staff not following correct cleaning procedures than to do with disinfectant resistance. Control of the outbreak is more likely to occur when the source is identified and eliminated. Hydrogen peroxide vapor can also be used as an effective mode of decontamination.^8^

In conclusion, *Acinetobacter baumannii* can be found as a colonizing bacterium with no adverse effects. However, it can be contaminated easily within a hospital or battlefield environment, and when it becomes an infective organism, it may be difficult to treat, as multidrug resistance is common. Those who are immunocompromised, have chronic disease, have surgical wounds, or have prolonged hospital admissions are particularly vulnerable. When a patient is proven to be colonized with *Acinetobacter*, the patient should be isolated and appropriate infection control measure put in place to prevent colonization or infection of other patients.
